# Combinations of Ashwagandha Leaf Extracts Protect Brain-Derived Cells against Oxidative Stress and Induce Differentiation

**DOI:** 10.1371/journal.pone.0120554

**Published:** 2015-03-19

**Authors:** Navjot Shah, Rumani Singh, Upasana Sarangi, Nishant Saxena, Anupama Chaudhary, Gurcharan Kaur, Sunil C. Kaul, Renu Wadhwa

**Affiliations:** 1 Cell Proliferation Research Group and DBT-AIST International Laboratory for Advanced Biomedicine (DAILAB), National Institute of Advanced Industrial Science & Technology (AIST), Tsukuba 305-8562, Japan; 2 Department of Biotechnology, Guru Nanak Dev University, Amritsar 143005, India; Indiana University, UNITED STATES

## Abstract

**Background:**

Ashwagandha, a traditional Indian herb, has been known for its variety of therapeutic activities. We earlier demonstrated anticancer activities in the alcoholic and water extracts of the leaves that were mediated by activation of tumor suppressor functions and oxidative stress in cancer cells. Low doses of these extracts were shown to possess neuroprotective activities *in vitro* and *in vivo* assays.

**Methodology/Principal Findings:**

We used cultured glioblastoma and neuroblastoma cells to examine the effect of extracts (alcoholic and water) as well as their bioactive components for neuroprotective activities against oxidative stress. Various biochemical and imaging assays on the marker proteins of glial and neuronal cells were performed along with their survival profiles in control, stressed and recovered conditions. We found that the extracts and one of the purified components, withanone, when used at a low dose, protected the glial and neuronal cells from oxidative as well as glutamate insult, and induced their differentiation *per se*. Furthermore, the combinations of extracts and active component were highly potent endorsing the therapeutic merit of the combinational approach.

**Conclusion:**

Ashwagandha leaf derived bioactive compounds have neuroprotective potential and may serve as supplement for brain health.

## Introduction

Ashwagandha (*Withania somnifera*; Solanaceae) is one of the most commonly used herbs in Ayurveda, an Indian system of traditional home medicine in practice for thousands of years to sustain general health spectrum on one hand, and to treat many kinds of disorders on the other. It is widely regarded as the number one adaptogenic tonic. The major constituents of Ashwagandha extracts, from various parts of the plant including leaves, shoots and roots, are withanolides (steroidal alkaloids and lactones with ergaostane skeleton) [1] of which withanone, withaferin A, withanolide A and withanolide D constitute the major fractions, and have recently been studied for their anticancer, immunomodulatory and neuroregenerative activities. Anticancer activity of withaferin A and withanone has been shown to be mediated by activation of tumor suppressor proteins, induction of oxidative stress and its deteriorating effects on cell skeleton and metabolism [2–16]. Withanolides were shown to stimulate cell-mediated immunity in several models including drug-induced myelosuppression in mice [17,18]. Withaferin A was proposed as a natural anti-inflammatory agent based on its inhibitory activity on IkappaB phosphorylation and degradation causing cytoplasmic retention of NFkappaB and hence inhibition of its transcriptional activation function [19]. On the other hand, it was shown that the phytochemicals from Ashwagandha could scavenge free radicals generated by gentamicin and result in recovery of gentamicin-induced nephrotoxicity and liver function in mouse model [20]. Alcoholic extracts of Ashwagandha leaves offered protection against scopolamine-induced amnesia by recovering cholinergic blockade and oxidative stress in brain and brain-derived cells, as determined by increase in expression of BDNF, GFAP, ARC, NF-200, MAP2, PSD-95 and GAP-43 [21,22]. Low doses of alcoholic and water extracts of Ashwagandha were shown to induce differentiation in glial and neuroblastoma cells [23–26], and hence proposed as natural differentiation inducing therapeutic agents for brain cancer. Taken together, experimental evidence suggested potentials of Ashwagandha phytochemicals for cancer therapy as well as treatment of neurodegenerative disorders. However, comparative study on the therapeutic potential, such as neuroprotective effect, in alcoholic and water extracts has not been reported.

Glutamate, a major excitatory neurotransmitter in the central nervous system, is involved in brain functions including cognition, memory and learning. It articulates signaling network that regulates brain development, differentiation and functioning of synapses. Whereas an optimal dose of glutamate is essential for normal brain physiology, its low and high doses trigger neurotoxic or excitotoxic cascades [27–29]. Its physiological and pathological effects are mediated mainly via two types of ionotropic glutamate receptors, the NMDA (N-methyl-D-aspartate) receptor and the non-NMDA (α-amino-3-hyroxy-5-methylisoxazole proprionic acid (AMPA)) or metabotropic (mGlu1-mGlu8) receptors [30,31]. Activation of NMDA receptors by pathologically high level of glutamate causes influx of extracellular Ca^2+^ leading to activation of number of enzymes that lead to neuronal death (called excitotoxicity) [29,32], a common feature of neurodegenerative disorders in the central nervous system (CNS) including Alzheimer’s disease (AD), Parkinson disease (PD), amyotrophic lateral sclerosis (ALS) and multiple sclerosis (MS) [27,33]. Excitotoxicity is also involved in spinal cord injury, stroke and trauma and cause oxidative stress, an imbalance in the generation and disposal of ROS [34].

Oxidative stress has been implicated and closely connected to neurodegenerative disorders [35–37]. Molecular mechanisms of glutamate-induced neurotoxicity and excitotoxicity have not been completely understood. They are frequently linked to oxidative stress as the free radical-scavenging agents, and antioxidants, such as vitamin E, have been shown to have protective impact on glutamate-toxicity [38]. We had earlier reported that the alcoholic extract of Ashwagandha leaves (i-Extract) protects normal human fibroblasts against oxidative stress caused by hydrogen peroxide and an industrial toxin, methoxyacetic acid (MAA) [39,40]. In the present study, we investigated the therapeutic potential of leaf extracts against oxidative stress and glutamate-toxicity in brain derived cells. We demonstrate that the (i) alcoholic (i-Extract) and water (WEX) extracts of Ashwagandha leaves cause protection against oxidative stress and glutamate toxicity. The extracts induce differentiation in glioblastoma and neuroblastoma cells, (ii) combinations of the active components from two types of extracts were highly effective.

## Results

### Ashwagandha leaf extracts protected neuroblastoma and glioblastoma against oxidative- and DNA damage-stress caused by hydrogen peroxide

**Fig 1:**
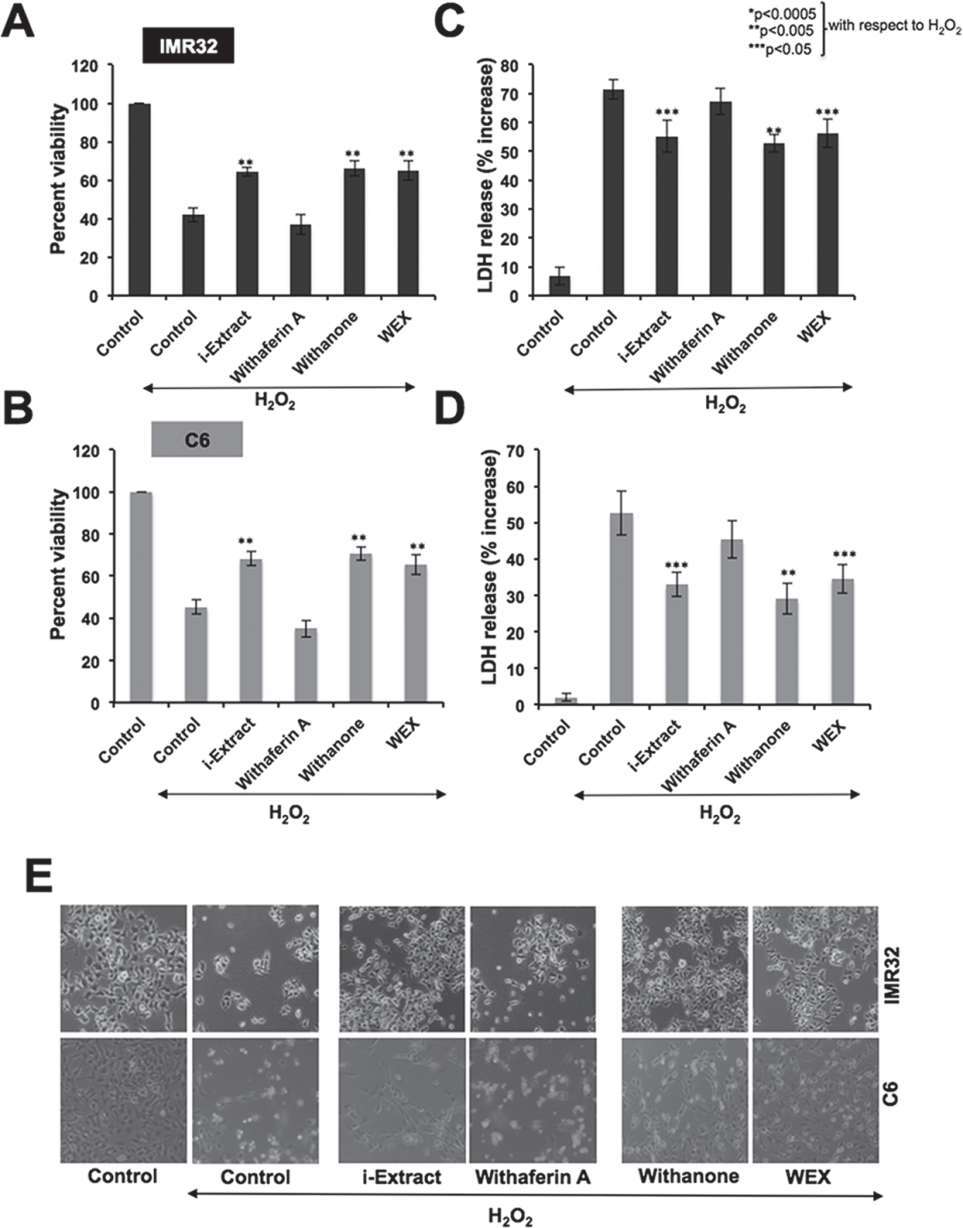
Cell viability (A and B) and cell death (C and D) as determined by MTT and LDH assays, respectively, in human neuroblastoma (IMR32) and rat glioblastoma (C6) cells treated with H_2_O_2_ and recovered either in the absence (control) or presence of Ashwagandha reagents-supplemented medium is shown. Morphology of control and treated cells is shown in E. Values are presented as mean ± SEM of three independent experiments. Statistical significance (P values) between the control and H_2_O_2_-treated group recovered either in i-Extract, (1 μg/ml for C6 and 0.4 μg/ml for IMR32), withaferin A (0.3 μg/ml for C6 and 0.2 μg/ml for IMR32), withanone (5 μg/ml for C6 and 2 μg/ml for IMR32) and WEX 1% supplemented medium for 24 h are shown. Statistical significance with P<0.05 was considered significant.

Human neuroblastoma (IMR32) and rat glioblastoma (C6) cells, pretreated with Ashwagandha-reagents for 24 h, were exposed to H_2_O_2_ (300 μM) for 2 h. The cells were then incubated in control or test medium supplemented with either the alcoholic (i-Extract) or water (WEX) extracts or their active components (withanone or withaferin A) as indicated. After 24 h of recovery, MTT assays revealed that the cell viability was reduced to about 40% in response to the H_2_O_2_ treatment. On the other hand, recovery of cells in the presence of i-Extract, withanone or WEX resulted in an increase in viability by 20–30%. Cells recovered in the presence of withaferin A-supplemented medium did not show protective effect for both C6 and IMR32 cells (Fig. 1A and 1B). The data was further confirmed by LDH assay. Cells exhibited increase in LDH release when treated with H_2_O_2_, implicating its cytotoxic effect. As shown in Fig. 1C and 1D, LDH release was significantly reduced in cells when recovered in the presence of either i-Extract or withanone or WEX in the culture medium. Similar to the viability assay, LDH assay also showed least protective effect in case of withaferin A-treated cells. Furthermore, cell morphology endorsed that the i-Extract, withanone and WEX protected IMR32 and C6 cells against H_2_O_2_ stress (Fig. 1E).

**Fig 2:**
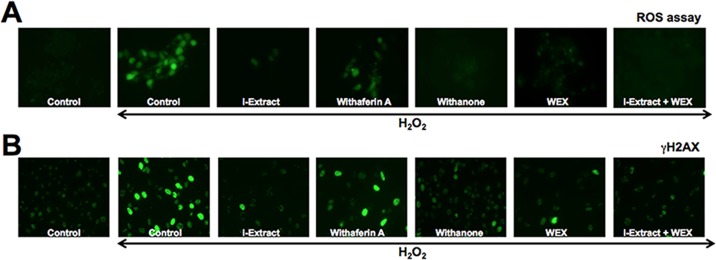
Oxidative stress and DNA damage response as determined by staining of Reactive Oxygen Species (A) and γH2AX (B) in control and H_2_O_2_ treated C6 cells. Whereas H_2_O_2_ treated cells showed increase in ROS and γH2AX, cells recovered either in the presence of i-Extract, withanone, WEX or combination of i-Extract and WEX showed decrease.

In order to investigate whether i-Extract, withanone and withaferin A protected the cells against oxidative or DNA damage stress, we examined the level of ROS and γH2AX, respectively, in cells recovered in control and test reagents (as indicated). As shown in Fig. 2A, H_2_O_2_ treated C6 cells showed increase in ROS as well as γH2AX suggesting that it caused both oxidative and DNA damage stress. The cells recovered in test medium containing either i-Extract or withanone or WEX showed significant reduction in ROS as well as γH2AX implying their protective effect. Cells recovered in withaferin A-supplemented medium showed least recovery based on the level of expression of either ROS or γH2AX. Similar results were obtained for IMR32 cells (data not shown).

### Ashwagandha leaf extracts instigated protection of differentiated glial and neuronal cells against H_2_O_2_ stress

**Fig 3:**
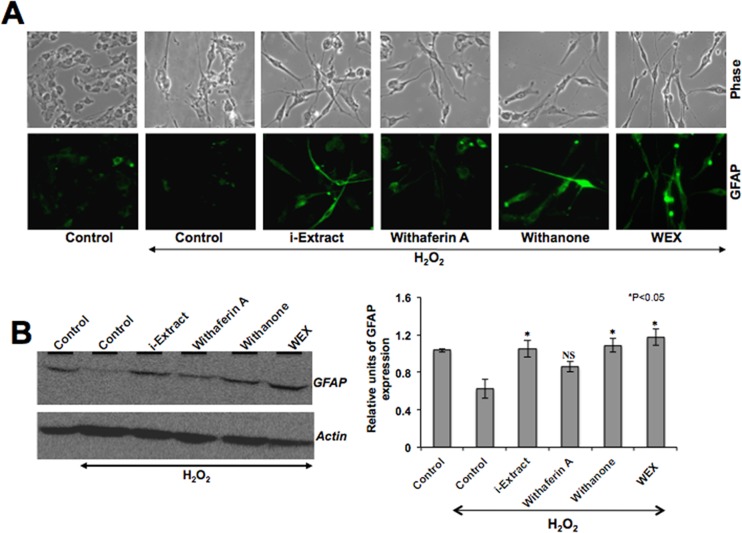
Morphology (A) and GFAP protein level (B) of C6 glioblastoma cells treated with H_2_O_2_ and recovered in either control or Ashwagandha reagents (as indicated) supplemented medium. Whereas the level of GFAP expression decreased in cells stressed with H_2_O_2_ treatment, it increased in cells recovered in the presence of i-Extract, withanone and WEX. Quantitation of the Western blot is shown on the right. Statistical significance with P<0.05 was considered significant. NS denotes not significant.

**Fig 4:**
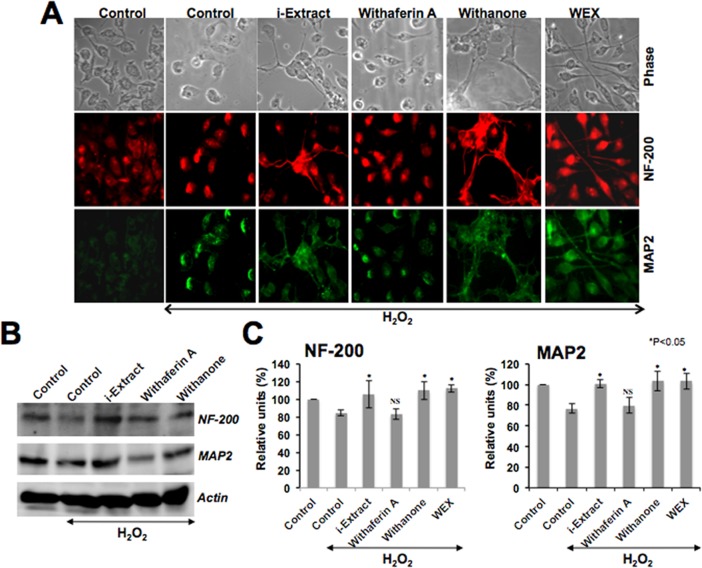
Morphology and immunofluorescence of NF-200 and MAP2 (A) and expression level of these proteins (B) in IMR32 neuroblastoma cells treated with H_2_O_2_ and recovered in the absence (control) or presence of Ashwagandha reagents in the medium. Quantitation of the protein levels as determined by three independent experiments is shown in C. Statistical significance with P<0.05 was considered significant. NS denotes not significant.

Based on the protection of oxidatively-challenged glioblastoma and neuroblastoma by Ashwagandha extracts as described above (Figs. 1 and 2), we next examined whether these phytochemicals could protect the differentiated C6 and IMR32 cells against the oxidative stress. The cells were first differentiated by retinoic acid (RA) treatment as described in the material and methods and then exposed to H_2_O_2_ followed by their recovery in the test medium as indicated. As shown in Fig. 3A, the cells recovered in either i-Extract or withanone or WEX-supplemented medium showed differentiated cell morphology that was further confirmed by immunostaining with glial cell marker, GFAP (Fig. 3A). Immunoblotting assay revealed that the level of GFAP expression was downregulated in H_2_O_2_-challenged cells but recovered when these stressed cells were cultured in either i-Extract- or withanone- or WEX-supplemented medium. Cells recovered in withaferin A-supplemented medium showed an insignificant increase in GFAP as observed by Western blotting and immunostaining (Fig. 3A and 3B). Similar to the differentiated glial cells, RA-differentiated neuronal cells when exposed to oxidative stress by exposure to H_2_O_2_ showed toxicity and death as seen in Fig. 4A. Cells recovered in the test medium as indicated (Fig. 4A) showed differentiated cell morphology in the presence of i-Extract or withanone or WEX. Based on the morphological observations and the expression levels of the neuronal differentiation markers, NF-200 (axonal marker) and MAP2 (dendritic marker), withaferin A-treated cells showed least protection (Fig. 4A and 4B).

### Ashwagandha leaf extracts protected the differentiated state in glutamate-challenged glial and neuronal cells

**Fig 5:**
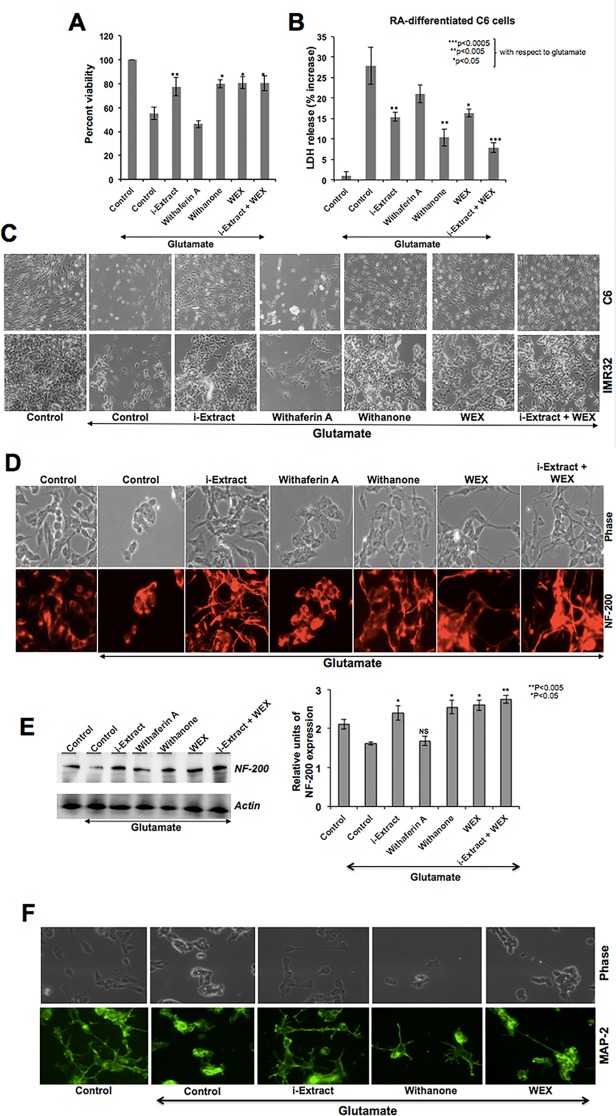
Cell viability (A) and cell death (B) as determined by MTT and LDH assays, respectively in C6 glioblastoma treated with glutamate and recovered either in the absence (control) or presence of Ashwagandha reagents-supplemented medium is shown. Morphology of control and treated C6 and IMR32 cells is shown in C. Immunostaining of control and treated IMR32 cells for NF-200 and immunoblotting showing its level of expression are shown in D and E, respectively. Quantitation is shown on the right. Statistical significance with P<0.05 was considered significant. NS denotes not significant. Immunostaining of cells for MAP2 showing protection of the differentiation state in cells in the presence of Ashwagandha reagents is shown in F.

**Fig 6:**
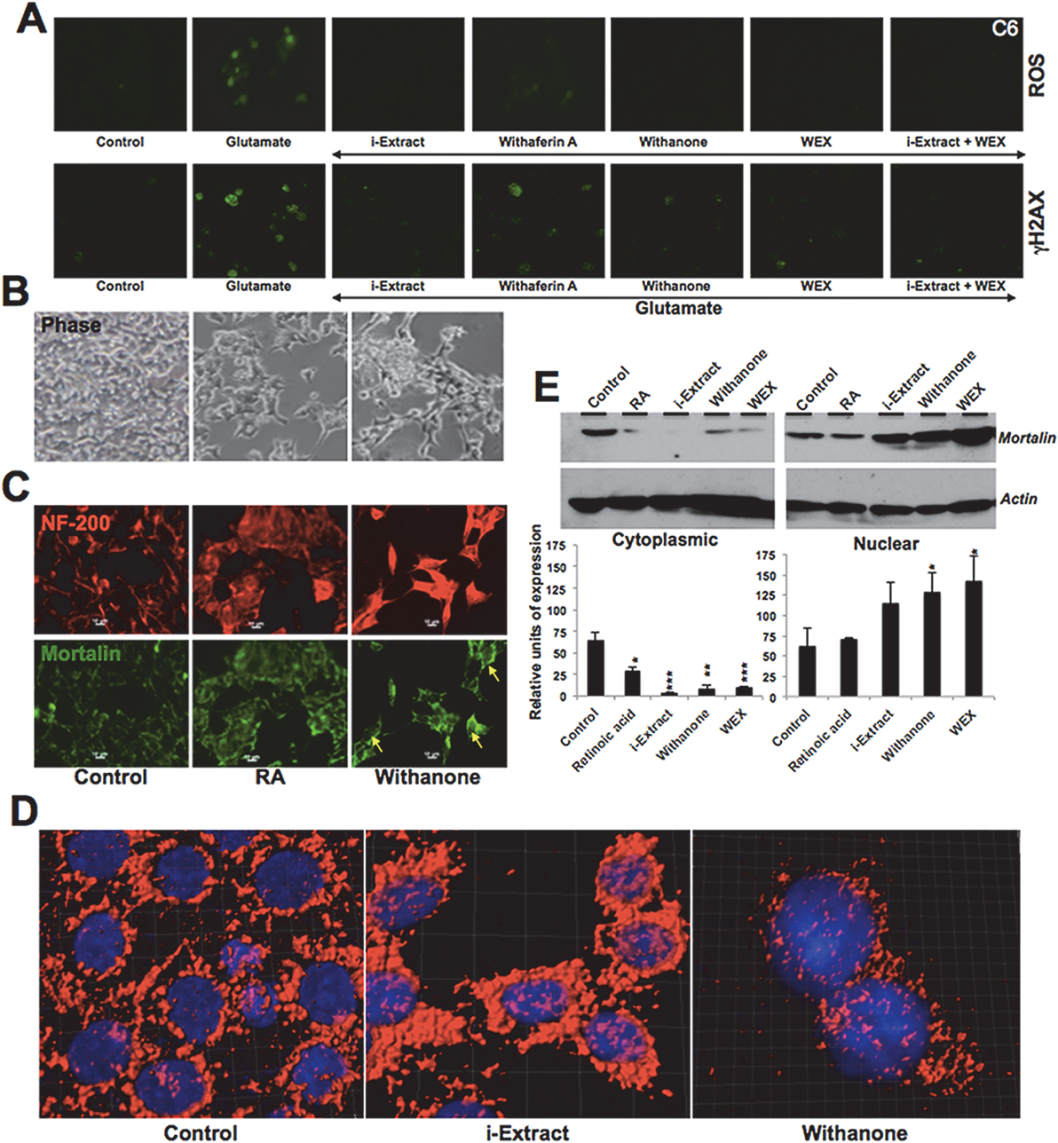
Induction of oxidative stress (ROS staining) and DNA damage response (γH2AX) in cells challenged with glutamate followed by their recovery in medium supplemented with Ashwagandha reagents (A) are shown. IMR32 cells treated with withanone showed differentiated morphology (B) and molecular markers of differentiation including increase in NF-200 (C) and nuclear translocation of mortalin (C, D and E). (C) RA-induced neuronal differentiation was used as a control. Along with the increase in NF-200, RA-treated cells showed presence of mortalin in the nucleus (also indicated by yellow arrows). (D) High-resolution confocal images showing mortalin staining (red). In the i-Extract- and Withanone-treated cells mortalin is prominent in the nucleus. Nuclei were stained with Hoechst (blue). (E) Detection of mortalin in the nuclear fractions by Western blotting. Decrease in the cytoplasmic, and increase in the nuclear fractions were observed in cells treated with i-Extract, Withanone and WEX. Actin was used as a loading control. Quantitation and statistical significance are shown.

Based on the above data, we investigated the effect of Ashwagandha extracts on glutamate-induced cytotoxicity in differentiated glial and neuronal cells. Cells were differentiated with retinoic acid for 5 days, followed by incubation in Ashwagandha reagents for 24 h, and then challenged with glutamate (250 μM for IMR32 and 500 μM for C6) for 24 h. As shown in Fig. 5A, cells recovered in either i-Extract or withanone or WEX-supplemented medium showed higher viability as compared to the control cells. Furthermore, combination of i-Extract and WEX resulted in enhanced recovery. In agreement with the increase in viability, LDH release significantly decreased when cells were recovered in i-Extract or withanone or WEX-supplemented medium. The LDH release was lowest in the presence of i-Extract and WEX combination. As shown in Fig. 5C, glutamate-treated cells were rounded and with shrunken morphology. The cells pretreated and recovered in the presence of i-Extract, withanone, WEX or a combination of i-Extract and WEX showed considerable protection. We examined the expression of neuronal differentiation markers and found that the expression of both NF-200 and MAP2 was protected in i-Extract-, withanone- or WEX-supplemented medium (Fig. 5, D-F). We also investigated if such protection of the differentiated state of cells was (i) associated with protection against the oxidative and DNA damage or (ii) associated with increase in differentiation potential of cells *pre se*. As shown in Fig. 6A, there was an increase in ROS and γH2AX in glutamate-challenged cells. The cells recovered in medium supplemented with Ashwagandha extracts (as indicated in Fig. 6) showed recovery from both oxidative and DNA damage stress. Of note, similar to the findings on maintenance of differentiation stage, i-Extract, withanone and WEX treated cells showed better recovery than the cells treated with withaferin A alone suggesting that the maintenance of differentiation was associated with protection against the oxidative and DNA damage stress. Furthermore, the combination of i-Extract and WEX showed best recovery as supported by very low levels of ROS and γH2AX, and high levels of differentiation marker protein, NF-200 (Figs. 5, D-E and 6A). In order to investigate the induction of differentiation by Ashwagandha-extracts *per se*, we used retinoic acid (RA)-induced differentiation as control and examined the expression of NF-200. As shown in Figs. 5D and 6C, i-Extract, WEX, withanone and RA-treated cells showed increase in NF-200. Of note, the RA-differentiated cells showed the nuclear localization of mortalin in agreement with the report by Shih et al [41] that demonstrated mortalin as an essential and early marker of neuronal differentiation. Furthermore, similar to the RA-differentiated cells, i-Extract and withanone-treated cells showed presence of mortalin in the nucleus by immunostaining (Fig. 6C and 6D) as well as immunoblotting of cell fractions (Fig. 6E). Taken together, the above data suggested that the Ashwagandha reagents induce differentiation in neuroblastoma cells.

## Discussion

Ashwagandha leaf extracts have earlier been shown to possess anticancer activities [2–4, 24, 42] in a variety of human cancer cells. Whereas high doses of the alcoholic and water extracts were shown to cause apoptosis or growth arrest of cancer cells, the low doses were shown to cause differentiation, and hence were proposed as candidate natural drugs for differentiation based therapy [23,26]. Low doses of withanone and water extract were also shown to protect against scopolamine-induced loss of memory function [21,22] and glutamate-induced excitotoxicity [25], respectively. Comparative study on the effect of alcoholic and water extract in response to oxidative stress (a prime cause of neurodegenerative diseases) and in brain-derived differentiated cells has not been reported. We addressed these issues in the present study. C6 (glioblastoma) and IMR32 (neuroblastoma) cells were subjected to oxidative stress followed by recovery in medium-supplemented with either the alcoholic or water extract showed protection against stress and maintained the differentiation state. It has been reported that in rats, the Ashwagandha extracts exert *in vivo* neuroprotection against stress, and is due to the antioxidant properties of its constituents [43]. In cell-based assays, we examined the effect of Ashwagandha extracts on established markers of oxidative stress (ROS) and DNA damage (H2AX). It has been established that in mammalian cells, phosphorylation of H2AX at Ser139 occurs in response to DNA double-strand breaks. The phosphorylated form of H2AX (γH2AX) along with other DNA damage response proteins (ATM, ATR, CHK-1 and CHK-2), constitute DNA damage foci in the nucleus that are easily identified by immunostaining with anti-H2AX antibody [44]. These assays revealed that Ashwagandha extracts caused reduction in H_2_O_2_- and glutamate-induced accumulation of ROS and γH2AX, suggesting that the neuroprotection was mediated, at least in part, by their anti-oxidative properties. We found that the protective effect of the alcoholic and the water extracts was comparable. Furthermore, whereas withanone was protective against oxidative stress, withaferin A was not effective, at least, at the doses used in the present study. In order to evaluate the therapeutic potential of these extracts for neurodegenerative diseases, we used differentiated glial and neuronal cells and subjected them to glutamate cytotoxicity, an established cause of neurodegeneration and decline in memory functions [30]. We found that the glutamate-induced oxidative stress and DNA damage to differentiated glial and neuronal cells were inhibited when these cells were recovered in i-Extract, withanone or WEX-supplemented medium. The combination of i-Extract and WEX showed better recovery. The cells showed increase in their survival capacity, reduced accumulation of ROS and γH2AX foci formation (indicative of DNA damage response) and maintenance/induction of differentiation. Either H_2_O_2_- or glutamate-induced oxidative stress lead to reduction in GFAP (glial cell differentiation marker), NF-200 (axonal marker) and MAP2 (dendritic marker) signifying its impact on the major cytoskeletal components (myelinated axons and microtubules), essential for differentiated neurons. Chronic restraint stress to rats has also been reported to alter the expression and distribution of MAP2 in cortex and hippocampus [45]. Of note, in the present study, the cells treated with either i-Extract, withanone or WEX showed increase in GFAP, NF-200, MAP2 proteins, endorsing the protection and maintenance of functional state of both the glial and neuronal cells. These data suggested that the extracts of Ashwagandha and their components possess neuro-protective and neuro-differentiating potential, likely to be mediated by activation of NF-200 and MAP2 signaling. We found that withanone was more potent than withaferin A in all the assays, and was not toxic to the differentiated cells *per se*. Furthermore, the combination of i-Extract and WEX showed better protection in almost all assays suggesting that they may operate by independent pathways and hence a combination proves to have beneficial outcome. It has been shown that the alcoholic and water extract of leaves have distinct constituents. Withaferin A and withanone are present in the alcoholic, but not water, extract; the latter was characterized to possess triethylene glycol [2–4, 42]. Therefore, it is likely that the better protection by combination treatment is due to the additive effect of the active components that may work by independent pathways. Molecular characterization of these pathways warrants further studies. We also found that the i-Extract, WEX and withanone induce differentiation in neuroblastoma cells *per se*, as endorsed by nuclear translocation of mortalin that has been shown to play an essential role in neuronal differentiation [41]. Interestingly, nuclear mortalin, in the absence of retinoic acid (RA), in cancer cells was shown to enhance their malignant properties by inactivating p53 and activating telomerase and hnRNP-K proteins [46]. In RA-treated neuroblastoma, mortalin was shown to translocate into nucleus, bind to retinoic acid receptors (RAR) causing reduction in their proteasome-mediated degradation and hence augment their recruitment to the retinoic acid response element (RARE) for transcriptional activation of downstream effector genes involved in neuronal differentiation. Knockdown of mortalin was shown to cause a significant decrease in RA-triggered gene expression implicating a novel function of nuclear mortalin in actively promoting neuronal differentiation [41]. Similar to the effect of RA, i-Extract or withanone treatment was seen to cause nuclear enrichment of mortalin in IMR32 cells implying that these phytochemicals have neuro-differentiation potential. Based on these findings, such as (i) protection against oxidative stress, DNA damage and glutamate excitotoxicity, (ii) maintenance and induction of differentiation, Ashwagandha leaf extracts and withanone are proposed as potent natural neurotherapeutic drugs. Further studies are warranted to resolve the signaling pathways and mechanisms involved in therapeutic potential of individual and combination of these extracts and phytochemicals.

## Material and Methods

### Preparation of the extracts

Extracts were prepared from the dried Ashwagandha leaves, as described previously (2, 42). Their activities were tested by cell viability assays as described below.

### Cell culture

Brain-derived glioma C6 (rat) and neuronal IMR32 (human) cell lines were obtained from Cell Resource Center for Biomedical Research, Tohoku University, Japan. C6 cells were maintained in Dulbecco’s Modified Eagle’s Medium (DMEM; Invitrogen)-supplemented with 10% fetal bovine serum. IMR32 cells were maintained in Minimal Essential Medium (MEM)-supplemented with 10% fetal bovine serum and 1% NEAA-nonessential amino acids in a humidified incubator (37°C and 5% CO_2_). Equal number of cells was plated in 6-well plates (50,000/well) or coverslips placed in 12-well (10,000/well) plates for biochemical or visual detection of the indicated protein markers. Cells were grown to achieve 60% confluency and then treated with low doses of alcoholic extract of Ashwagandha leaves (i-Extract, 1 μg/ml for C6 and 0.4 μg/ml for IMR32), withaferin A (0.3 μg/ml for C6 and 0.2 μg/ml for IMR32) and withanone (also called i-Factor, 5 μg/ml for C6 and 2 μg/ml for IMR32) for 24 h. These concentrations were determined from the independent assays on cell viability and determination of IC50 values as described earlier (2, 3, 4, 23 and 24). The non-toxic doses were selected. The cells pretreated with the indicated reagents for 24 h, were stressed with H_2_O_2_ (300 μM in C6 and 150 μM in IMR32) for 1 to 2 h followed by their recovery in medium supplemented with indicated reagents as described above. Pre-treatment regime was selected based on independent experiments, including addition of Ashwagandha reagents to cell culture medium 24 h prior (pre-treatment)/ along with (co-treatment) or after (post-treatment) the stress (as described below) followed by recovery (as indicated). We found that the pre-treatment was most effective.

For differentiation model, IMR32 or C6 cells were cultured in a medium supplemented with 10 μM retinoic acid for 5 days with change of medium every alternate day followed by treatment with Ashwagandha reagents in retinoic acid medium as described above for 24–48 h. The cells were then exposed either to glutamate (250 μM in IMR32 and 500 μM in C6) for overnight or H_2_O_2_ (300 μM for C6 and 150 μM for IMR32 cells) for 1–2 h followed by recovery (24 h) in Ashwagandha components-supplemented medium. Cells were then harvested for molecular and imaging assays as described below.

### Cell viability assay

Cell viability was measured by the quantitative colorimetric MTT [3-(4, 5-dimethylthiazol-2-yl)-2,5-diphenyltetrazolium bromide] assay showing the mitochondrial activity of living cells. Briefly, assays were performed in 96-well plates (5000 cells/well). Cells were stressed and recovered as described above. MTT (0.5 mg/ml) was added to the cell culture medium at the end of treatments and incubated for 4 h in a humidified incubator (37°C and 5% CO_2_). After incubation, the supernatant was carefully removed and the crystals were dissolved in 100 μl DMSO. The cell viability was quantitated by the conversion of yellow MTT reagent by mitochondrial dehydrogenases of living cells to purple formazan. The absorbance was measured at 550 nm using spectrophotometer (Wallac, ArvoSX). Experiments were done in triplicates and the standard deviation was estimated from three independent experiments. Statistical significance of the data was determined by unpaired t-test using GraphPad software.

### LDH-cytotoxicity assay

Cell membrane damage/cytotoxicity was determined using LDH release assay. Quantitative measure of LDH (a stable cytosolic enzyme, present in all cell types that is rapidly released upon cell membrane damage or cell lysis) in the culture supernatants provides a reliable measure of cytotoxicity. C6 and IMR32 cells (at about 60% confluency) were treated with indicated Ashwagandha-reagents in 96-well plates. Culture supernatants were then collected from each well and assayed for LDH release following the manufacturer’s instructions (Abcam). The absorbance was measured at 450 nm using a microplate reader (Wallac, ArvoSX). The percent cytotoxicity was calculated with reference to untreated and treated control.

### Detection of reactive oxygen species

The reactive oxygen species were detected by fluorescence staining using the Image-iT LIVE Green Reactive Oxygen Species (ROS) Detection Kit (Molecular Probes Inc., USA). Cells (10,000/well) were plated on glass coverslips placed in 12-well plates. At about 60% confluency, cells were treated with Ashwagandha reagents for 48 h followed by stress conditions and recovery as indicated. Cells were stained for ROS following the procedure recommended by the manufacturers.

### Immunofluorescence

Cells (10,000/well) were plated on glass coverslips placed in 12-well culture dish. At about 60% confluency, cells were stressed and recovered as indicated, followed by washings with cold phosphate-buffered saline (PBS) and fixation with pre-chilled methanol:acetone (1:1 v/v) mixture for 5–10 min. Fixed cells were washed with PBS, permeabilized with 0.2% Triton X-100 in PBS for 10 min, and blocked with 2% bovine serum albumin (BSA) in PBS for 20 min. Cells were stained with anti-GFAP (Sigma), anti-NF-200 (Sigma), anti-MAP2 (Sigma), anti-PSD-95 (Santa Cruz), anti-GAP43 (Santa Cruz) and anti-γH2AX (Millipore) antibodies. Immunostaining was visualized by secondary staining with either Alexa-488 or Alexa-594 conjugated antibodies (Molecular probes). After three to four washings with 0.2% Triton X-100 in PBS (PBST), cells were overlaid with Fluoromount (Difco). Stained cells were examined on a Zeiss Axiovert 200 M microscope and analyzed by AxioVision 4.6 software (Carl Zeiss). To examine the presence of mortalin in the nucleus, images were acquired with a confocal laser scanning microscope (Zeiss LSM 700). The files were transferred to a graphic work station and analyzed with Imaris software (Bitplane, Zurich, Switzerland).

### Cell fractionation

Cells (5x10^5^) were plated in 10-cm dishes. At about 70% confluency, they were treated as indicated above. Nuclear and cytoplasmic fractions were prepared using the Qproteome Cell Compartment kit following manufacturers instructions (Qiagen, Hilden, Germany). Fractions were examined for mortalin protein by immunoblotting, as described below.

### Immunoblotting

Cells (50,000 cells/well) were plated in 6-well plates and were grown to 60–70% confluency for treatments as described above. For Western blotting, cells were lysed with RIPA lysis buffer for 20 min on ice. Lysates were centrifuged at 15000 rpm for 15 min, and the supernatant (20 μg protein) was resolved on SDS-PAGE followed by transfer onto a nitrocellulose membrane (Millipore) using a semidry transfer blotter. Immunoassays were done with anti-GFAP (Sigma), anti-NF-200 (Sigma), anti-MAP2 (Sigma) and anti-actin (Chemicon International, Temecula, CA) antibodies. The immunocomplexes formed were visualized with horseradish peroxidase-conjugated anti-rabbit/mouse immunoglobulin G (IgG; ECL; Amersham Pharmacia Biotech, Piscataway, NJ).
